# Primary empathy deficits in frontotemporal dementia

**DOI:** 10.3389/fnagi.2014.00262

**Published:** 2014-10-10

**Authors:** Sandra Baez, Facundo Manes, David Huepe, Teresa Torralva, Natalia Fiorentino, Fabian Richter, Daniela Huepe-Artigas, Jesica Ferrari, Patricia Montañes, Pablo Reyes, Diana Matallana, Nora S. Vigliecca, Jean Decety, Agustin Ibanez

**Affiliations:** ^1^Institute of Cognitive Neurology (INECO) & Institute of Neuroscience, Favaloro UniversityBuenos Aires, Argentina; ^2^UDP-INECO Foundation Core on Neuroscience (UIFCoN), Diego Portales UniversitySantiago, Chile; ^3^National Scientific and Technical Research Council (CONICET)Buenos Aires, Argentina; ^4^Australian Research Council (ACR) Centre of Excellence in Cognition and its DisordersSydney, NSW, Australia; ^5^Laboratory of Cognitive and Social Neuroscience, Universidad Diego PortalesSantiago, Chile; ^6^Department of Psychology, University of CologneCologne, Germany; ^7^Departamento de Psiquiatría y Salud Mental, Facultad de Medicina, Centro de Memoria y Cognición Intellectus, Instituto de Envejecimiento, Universidad Javeriana, Hospital San IgnacioBogotá, Colombia; ^8^Universidad Nacional de ColombiaBogotá, Colombia; ^9^Instituto de Humanidades (IDH) de la Facultad de Filosofía y Humanidades, Universidad Nacional de CórdobaCórdoba, Argentina; ^10^Department of Psychology and Department of Psychiatry and Behavioral Neuroscience, University of ChicagoChicago, IL, USA; ^11^Universidad Autonoma del CaribeBarranquilla, Colombia

**Keywords:** behavioral variant of frontotemporal dementia, empathy, empathic concern, social cognition, executive functions, moral judgment

## Abstract

Loss of empathy is an early central symptom and diagnostic criterion of the behavioral variant frontotemporal dementia (bvFTD). Although changes in empathy are evident and strongly affect the social functioning of bvFTD patients, few studies have directly investigated this issue by means of experimental paradigms. The current study assessed multiple components of empathy (affective, cognitive and moral) in bvFTD patients. We also explored whether the loss of empathy constitutes a primary deficit of bvFTD or whether it is explained by impairments in executive functions (EF) or other social cognition domains. Thirty-seven bvFTD patients with early/mild stages of the disease and 30 healthy control participants were assessed with a task that involves the perception of intentional and accidental harm. Participants were also evaluated on emotion recognition, theory of mind (ToM), social norms knowledge and several EF domains. BvFTD patients presented deficits in affective, cognitive and moral aspects of empathy. However, empathic concern was the only aspect primarily affected in bvFTD that was neither related nor explained by deficits in EF or other social cognition domains. Deficits in the cognitive and moral aspects of empathy seem to depend on EF, emotion recognition and ToM. Our findings highlight the importance of using tasks depicting real-life social scenarios because of their greater sensitivity in the assessment of bvFTD. Moreover, our results contribute to the understanding of primary and intrinsic empathy deficits of bvFTD and have important theoretical and clinical implications.

## Introduction

Loss of empathy is an early symptom of behavioral variant of frontotemporal dementia (bvFTD) and constitutes one of its diagnostic criteria (Piguet et al., 2011; Rascovsky et al., 2011). Patients with bvFTD display a diminished response to other's feelings and a diminished social interest or personal warmth (Mendez, 2006; Rankin et al., 2006). From a clinical perspective, empathy changes influence the interpersonal judgment, emotions, behavior, and social functioning of bvFTD patients (Lough et al., 2006; Piguet et al., 2011; Rascovsky et al., 2011). In spite of its relevance, the study of empathy in bvFTD patients using experimental designs has been scarce, and no studies have explored whether relevant factors (Gregory et al., 2002; Possin et al., 2013) such as executive functions (EF) and other social cognition domains (OSCD) impact the empathic abilities of these patients.

Empathy is essential for human social interaction, comprising the capacity to share and understand the subjective experience of others in reference to oneself (Decety, 2011). This complex construct involves (1) affective components: sharing and responding to the emotional experience of others; (2) cognitive components: understanding the intentions and perspectives of others; and (3) aspects related to the moral evaluation: judgments about the wrongness of an action or the punishment that a perpetrator deserves (Decety and Jackson, 2004; Decety et al., 2012).

In spite of the complexity of empathy, traditional approaches to measure it have relied on self-report questionnaires. These questionnaires consider empathy as a trait and do not fully represent empathic abilities because of their limited ecological validity (Ickes, 2009). Nonetheless, most studies of empathy in bvFTD patients (Rankin et al., 2005, 2006; Lough et al., 2006; Eslinger et al., 2011) have employed self-report questionnaires, evidencing impairments in affective and cognitive components.

We implemented a novel paradigm with naturalistic stimuli that measures empathy for others' physical pain. This type of paradigm has been widely used due to the robustness of pain in inducing empathic responses (Bernhardt and Singer, 2012) and the well characterized neural circuit of empathy (Akitsuki and Decety, 2009). Here we employed an adaptation of an empathy for pain task (EPT) previously validated with behavioral measures, eye-tracking and fMRI (Decety et al., 2012). This adapted version has been used in the assessment of other neuropsychiatric populations (Baez et al., 2012, 2013; Baez and Ibanez, 2014). The task evaluates empathy in the context of intentional/accidental harms and consists of three different scenarios: (1) intentional or (2) accidental harms in which one person is in a painful situation intentionally or accidentally caused by another, and (3) neutral or control situations. The EPT evaluates the following components: (A) comprehension of the accidental or deliberate nature of the action and the intention of the perpetrator to hurt (cognitive components), (B) the empathic concern and the degree of discomfort for the victim (affective components), and (C) the correctness of the action and the punishment for the perpetrator (moral aspects).

Individual differences in empathy seem to be affected by two relevant factors: EF and OSCD. Some EF such as working memory (Ze et al., 2014), inhibitory control (Hansen, 2011; Ze et al., 2014), abstract reasoning and phonological fluency (Rankin et al., 2005) have been associated with self-report measures of cognitive empathy. Moreover, OSCD such as emotion processing (Singer, 2006), theory of mind (ToM) (Singer and Lamm, 2009) and moral cognition (Decety et al., 2012) have also been related to empathy. For instance, accurate recognition of facial emotion expressions is positively correlated with empathy (Besel and Yuille, 2010). Similarly, there is a positive correlation between ToM and empathy abilities (Shamay-Tsoory et al., 2007; Ibanez et al., 2013). Moreover, the relationship between empathy and morality is well established (e.g., Decety et al., 2012; Decety and Cowell, 2014; Escobar et al., 2014; Yoder and Decety, 2014). Empathy-related processes are thought to motivate prosocial behavior and caring for others, and to provide a foundation for morality (Decety et al., 2012; Decety and Cowell, 2014; Escobar et al., 2014). Empathy can also interfere with morality by introducing partiality, for instance by favoring ingroup members (Decety and Cowell, 2014). In addition, support for a link between empathy and moral cognition is provided by a recent study (Gleichgerrcht and Young, 2013) showing that low empathic concern levels predict utilitarian moral judgment.

On the other hand, it is well known that both EF (Viskontas et al., 2007; Torralva et al., 2009a; Possin et al., 2013) and social cognition (Gleichgerrcht et al., 2010; Ibanez and Manes, 2012) are impaired in bvFTD, but there are no studies exploring whether and how these factors affect the empathic abilities of these patients. This study assessed multiple empathy components in bvFTD patients by using an experimental paradigm involving ecological validity. We also employed several EF and OSCD (emotion recognition, ToM, and social norms knowledge) sensitive measures for the bvFTD assessment. Finally, we explored whether empathy deficits constitute a primary symptom of bvFTD or whether they are secondary to or a consequence from the EF or OSCD impairments.

## Methods and materials

### Participants

Thirty-seven patients fulfilled the Lund and Manchester criteria (Neary et al., 1998) and the revised criteria for probable bvFTD (Rascovsky et al., 2011) (see details regarding phenocopies or differential diagnoses in Supplementary Data). Patients presented with prominent changes in personality and social behavior as verified by caregivers. Diagnosis was made by a group of experts in bvFTD. Patients underwent a standard examination battery including neurological, neuropsychiatric and neuropsychological assessments and a routine MRI. All patients were in early/mild stages of the disease and did not meet the criteria for specific psychiatric disorders. Patients presenting primarily with language deficits were excluded. Of the 37 patients, 4 were excluded from the EPT analyses for inability to perform the task.

Thirty healthy controls were recruited and matched one by one with any of the bvFTD patients. Matching criteria were sex, age (±4 years) and years of education (±4 years) (see Table 1). Control subjects were recruited from a larger pool of volunteers who did not have a history of drug abuse or a family history of neurodegenerative or psychiatric disorders. All participants provided written informed consent in agreement with the Helsinki declaration. The Ethics Committee of the Institute of Cognitive Neurology approved this study.

**Table 1:** **Demographic, clinical and executive functions assessments**.

		**BvFTD (*n* = 37)**	**CTR (*n* = 30)**	**BvFTD vs. CTR**
Demographics	Age (years)	66.0 (7.43)	55.0 (8.64)	N.S.
	Gender (F:M)	15:22	15:15	N.S.
	Education (years)	13.68 (4.35)	14.67 (3.72)	N.S.
	MMSE	25.92 (3.53)	28.31 (1.54)	<0.01
Social Cognition	TASIT			
	Fear	2.26 (0.99)	3.27 (0.58)	<0.01
	Anger	3.18 (0.8)	3.7 (0.47)	N.S.
	Sadness	2.15 (1.1)	2.9 (0.61)	<0.01
	Surprise	3.5 (0.66)	3.83 (0.38)	N.S.
	Disgust	1.44 (0.96)	2.47 (0.94)	<0.01
	Total score	12.49 (2.74)	16.17 (1.56)	<0.01
	RMET	15.19 (5.24)	22.43 (4.95)	<0.01
	SNQ			
	Break score	2.35 (2.03)	2.04 (1.79)	N.S
	Over-adhere score	4.62 (1.71)	4.07 (2.11)	N.S
Executive functions	IFS Total Score	17.88 (6.15)	25.1 (1.87)	<0.01
	Motor series	2.54 (0.84)	2.97 (0.18)	<0.01
	Conflicting instructions	2.32 (0.97)	2.93 (0.25)	<0.01
	Go- no go	1.76 (1.14)	2.47 (0.51)	<0.01
	Backward digits span	3.43 (1.12)	4.37 (0.89)	<0.01
	Verbal Working memory	1.49 (0.69)	1.9 (0.31)	<0.01
	Spatial working memory	1.68 (0.88)	2.5 (0.94)	<0.01
	Abstraction capacity	1.53 (0.99)	2.7 (0.41)	<0.01
	Verbal inhibitory control	3.14 (2.02)	5.23 (0.68)	<0.01
	Phonological Fluency	10.88 (5.57)	16.3 (4.04)	<0.01
	Alternant design fluency	3.91 (2.01)	7.9 (2.4)	<0.01
	TMT-A	81.49 (48.26)	49.79 (23.08)	<0.01
	TMT-B	182.66 (93.22)	99.66 (52.44)	<0.01
	Hayling Test	21.68 (13.02)	9.31 (4.48)	<0.01

*IFS, INECO frontal screening; TMT, Trail making test; TASIT, The awareness of social inference test; RMET, Reading the mind in the eyes test; SNQ, social norms questionnaire*.

### Instruments

The cognitive state was assessed using the Mini-Mental State Examination (MMSE) (Folstein et al., 1983). The premobid intellectual level was evaluated by the word accentuation test (WAT-BA) (Burin et al., 2000).

#### Empathy assessment

**Figure 1:**
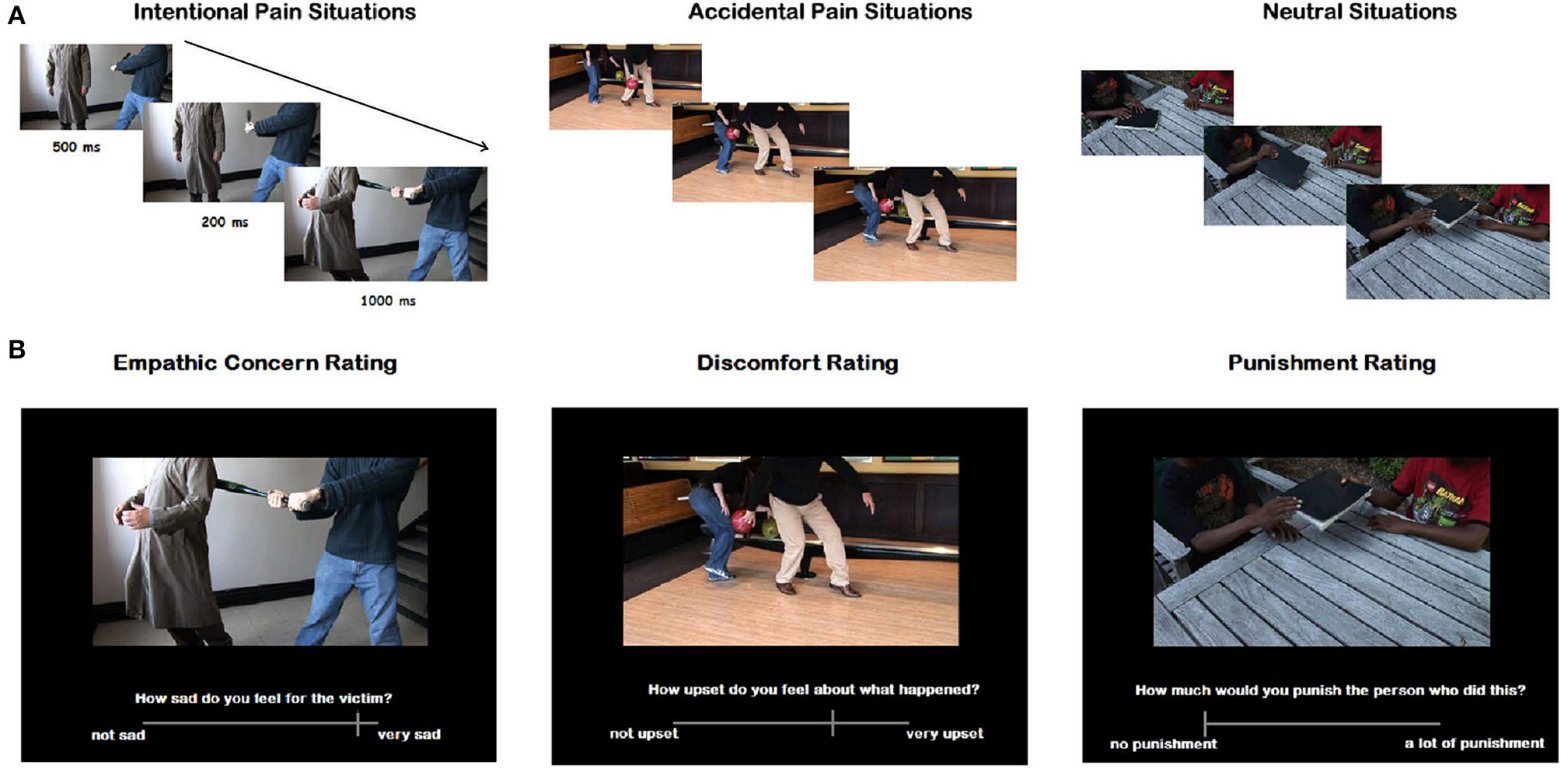
**(A)** Examples of the visual stimuli used for each category. The durations of the first, second, and third picture were 1000, 200, and 1000 ms, respectively. **(B)** Examples of the questions designed to assess different empathy aspects. Each question was answered using a computer-based visual analog scale.

We used an EPT previously employed in assessing other neuropsychiatric populations (Baez et al., 2012, 2013). This task evaluates empathy in the context of intentional and accidental harms (Baez et al., 2012, 2013) and consists of 25 animated scenarios (11 intentional, 11 accidental, 3 neutral) involving two individuals. Each scenario consists of 3 digital color pictures presented in a successive manner to imply motion. The durations of the first, second, and third pictures in each animation were 500, 200, and 1000 ms, respectively (see Figure 1 and Supplementary Movie 1). The three following types of situations were depicted: (1) intentional harm in which one person is in a painful situation intentionally caused by another, (e.g., purposely stepping on someone's toe); (2) accidental harm where one person is in a painful situation accidentally caused by another; and (3) control or neutral situations (e.g., one person receiving a flower given by another).

Importantly, the faces of the protagonists were not visible and thus there were no facial emotional reactions visible to participants. However, body expressions and postures provided sufficient information about the emotional reaction of the victim and the intention of the agent. Participants were asked to respond 6 different questions: (1) cognitive aspects of empathy: (a) intentionality (was the action done on purpose?) and (b) intention of the perpetrator to hurt the victim (how bad was the purpose?); (2) affective aspects: (c) emphatic concern (how sad do you feel for the victim?) and (d) degree of discomfort (how upset do you feel for what happened in the situation?); and (3) moral evaluation aspects: (e) correctness of the action (how inappropriate was the action?) and (f) punishment (how much penalty does this action deserve?). The question about the intentionality of the action was answered selecting “Yes/No.” The other questions were answered using a computer–based visual analog scale (it rates from −9 to 9, but the numbers were not visible to participants; see Movie 1). The meaning of the scale extremes depends on the question, for example on the question “how sad do you feel for the victim?” one extreme of the bar reads “I feel very sad” and the other extreme reads “I don't feel sad at all.” Accuracy for the intentionality question, and ratings as well as raw RTs for the other questions were measured. The RTs measured the time that passed from the moment the question appeared, to the time the participant answered.

Before testing, all participants performed a training session consisting in a shorter version of the task with similar situations, to ensure the correct understanding of the instructions.

#### Other social cognition domains (OSCD)

***Recognition of emotional states***

*The awareness of social inference test (TASIT)*. The TASIT is a test of social perception that involves videotaped vignettes of everyday social interactions (McDonald et al., 2003, 2006; Kipps et al., 2009b) which has been proved to be useful for detecting subtle deficits in bvFTD patients (Kipps et al., 2009a). This task introduces contextual cues (e.g., prosody, facial movement, and gestures) and additional processing demands (e.g., adequate speed of information processing, selective attention, and social reasoning) that are not taxed when viewing static displays. We considered only part 1, called the emotion evaluation test (EET), which assesses recognition of spontaneous emotional expression (fearful, surprised, sad, angry and disgusted). In the EET, speaker demeanor (voice, facial expression and gesture) together with the social situation indicate the emotional meaning. In some scenes, there is only one actor talking, who is either on the telephone or talking directly to the camera. Other scenes depict two actors and instructions are given to focus on one of them. All scripts are neutral in content and do not lend themselves to any particular emotion. The brief EET comprises a series of 20 short (15–60 s) videotaped vignettes of trained professional actors interacting in everyday situations. After viewing each scene, the test participant is instructed to choose from a forced-choice list the emotion expressed by the focused actor.

***ToM***

*Reading the mind in the eyes (RMET)*. This test (Baron-Cohen et al., 1997) evaluates the emotional inference aspect of the ToM and is another sensitive task for the assessment of bvFTD patients (Torralva et al., 2009a). This is a computerized and validated test which consist of 36 pictures of the eye region of a face. Participants are asked to choose which of four words best describes what the person in each photograph is thinking or feeling.

***Social norms knowledge***

*Social norms questionnaire (SNQ)*. We used a previously validated version (Baez et al., 2013) of the SNQ. This questionnaire consists of 22 yes-no questions, wich has been previously employed in the assessment of bvFTD patients (Possin et al., 2013). The participants were asked to determine whether a behavior would be appropriate in the presence of an acquaintance (not a close friend or family member) according to the mainstream culture. Two scores were derived. The break score was defined as the total number of errors made in the direction of breaking a social norm, and the over-adhere score was defined as the total number of errors made in the direction of over adherence to a perceived social norm.

#### Executive functions (EF)

All participants were evaluated with an EF battery which included the INECO frontal screening (IFS) (Torralva et al., 2009b) and measures of verbal fluency, inhibitory control, speed processing, working memory and cognitive flexibility. The IFS has been shown to successfully detect executive dysfunction in patients with dementia (Torralva et al., 2009b; Gleichgerrcht et al., 2011). This test includes the following eight subtests: (1) motor programming (Luria series, “fist, edge, palm”); (2) conflicting instructions (subjects were asked to hit the table once when the administrator hit it twice, or to hit the table twice when the administrator hit it only once); (3) motor inhibitory control; (4) numerical working memory (backward digit span); (5) verbal working memory (months backwards); (6) spatial working memory (modified Corsi tapping test); (7) abstraction capacity (inferring the meaning of proverbs), and (8) verbal inhibitory control (modified Hayling test). The maximum possible score on the IFS is 30 points.

Verbal and design fluency tests (Delis and Kaplan, 2001) were used to assess recall, self-monitoring and cognitive flexibility strategies. The trail-making test part B (Partington, 1949) was employed to assess cognitive flexibility and processing speed, and the Hayling test (Burgess and Shallice, 1996) was used to measure inhibitory control.

### Data analysis

Demographic and neuropsychological data were compared using one-way ANOVA and chi square tests for the categorical variables. The ratings and RTs for each question of the EPT were analyzed using 2 × 3 repeated-measures ANOVA comprising the factors of group (bvFTD, control) and condition (intentional, accidental, neutral). Tukey's HSD *post-hoc* tests were used (when appropriate) to examine group differences within each condition.

To control for general cognitive state on the EPT and OSCD performances, we applied ANCOVA tests adjusted for the MMSE scores (reporting only effects that were still significant after covarying). To determine whether empathy deficits were related to EF or OSCD, the empathy results were re-analyzed using the raw total scores of each measure of OSCD and EF independently as covariates [see for a similar approach (Rowe et al., 2001)]. These two analyses were conducted separately. The first one included all the EF total scores (IFS, verbal and design fluencies, TMT-B and Hayling test) as covariates, while the second one included all the OSCD measures (TASIT, RMET and SQN).

Finally, we conducted multiple regression analyses to explore whether empathy performance was partially explained by specific impairments in EF or OSCD. We estimated two different models in which the empathy measures that were still significantly different between groups after any of the covariance analyses were separately considered as dependent variables. The first model included a score of intentionality (the mean of the three conditions) as dependent variable; the second one considered as dependent variable the empathic concern scores for intentional pain situations. The group, the gender, a global score of OSCD (mean accuracy on TASIT and RMET) and the IFS total score were included as predictors. Gender was included as predictor since several studies (e.g., Baron-Cohen and Wheelwright, 2004; Toussaint and Webb, 2005; Preis and Kroener-Herwig, 2012) have reported that women show higher levels of empathy than males. We choose a global score of the OSCD from TASIT and RMET because in this and previous studies (Kipps et al., 2009a; Torralva et al., 2009a) detected bvFTD impairments. The IFS was also selected as a predictor because this tool includes several EF subtests and detects bvFTD executive dysfunction (Torralva et al., 2009b).

## Results

### Demographic data and general cognitive state

Groups were matched by age [*F*_(1, 65)_ = 0.10, *p* = 0.74], gender [*X*^2^_(1)_ = 0.59, *p* = 0.44], education [*F*_(1, 65)_ = 0.97, *p* = 0.32]. No differences between groups were observed in the premorbid IQ [*F*_(1, 65)_ = 1.54, *p* = 0.21]. As expected, bvFTD patients showed lower MMSE performance than controls [*F*_(1, 65)_ = 11.55, *p* < 0.01] (see Table 1).

To control for the effect of general cognitive state on empathy and OSCD performances, we applied ANCOVA tests adjusted for the MMSE scores. The empathy and OSCD results reported bellow correspond to the effects that were still significant after covariation.

### Empathy

**Figure 2:**
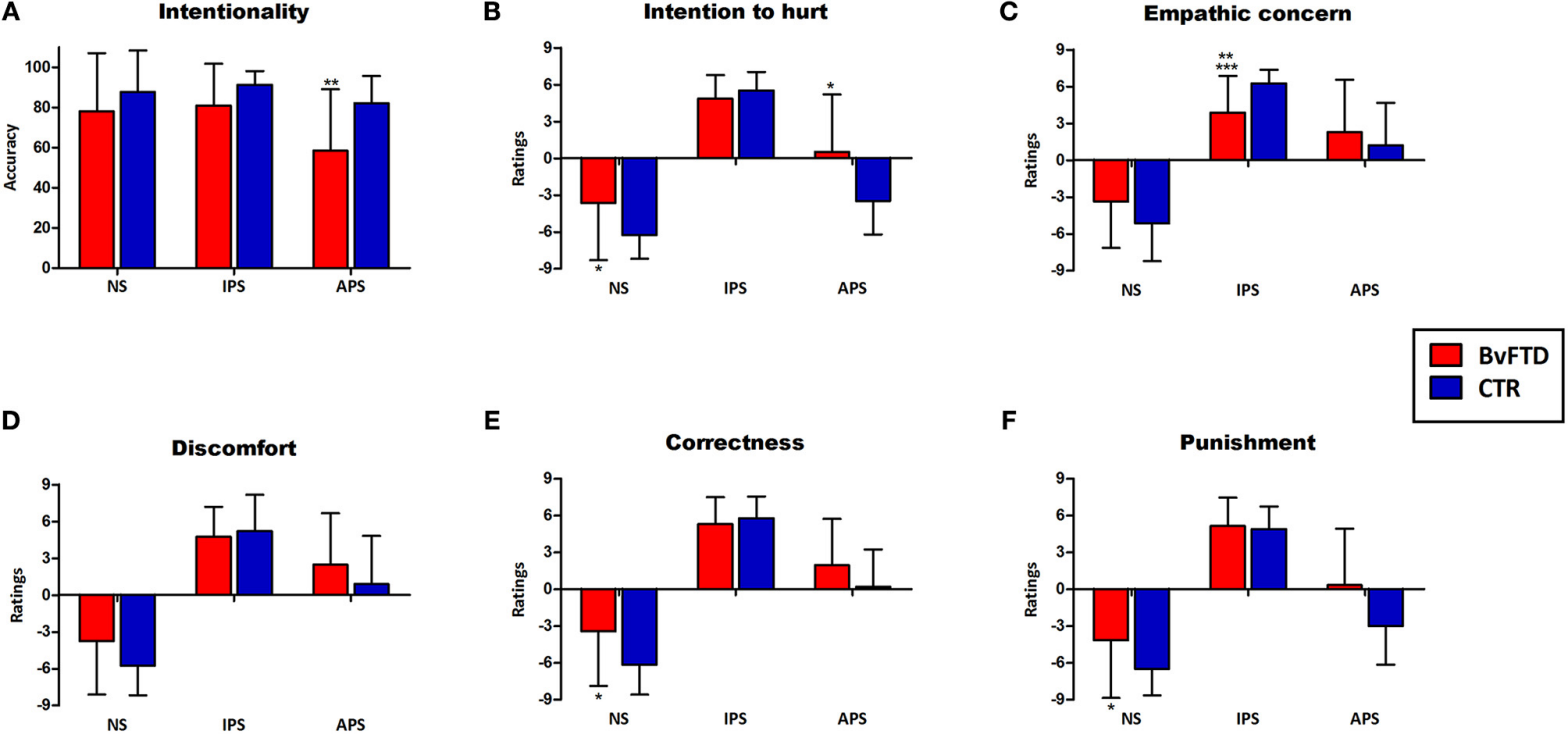
**Performance in the empathy for pain task and significant differences between groups**. Differences that were statistically significant are indicated by ^*^(before co-varying), ^**^(after co-varying by social cognition measures), and ^***^(after co-varying by EF). **(A)** Intentionality judgments; **(B)** Intention to hurt ratings; **(C)** Empathic concern ratings; **(D)** Discomfort ratings; **(E)** Correctness ratings; **(F)** Punishment ratings. NS, neutral situations; IPS, intentional pain situations, APS, accidental pain situations.

Results are summarized in Figure 2.

#### Cognitive components

Regarding intentionality comprehension, a main effect of condition was observed [*F*_(2, 122)_ = 7.43, *p* < 0.01, η^2^ = 0.11]. *Post-hoc* analysis (Tukey HSD, MS = 4.89, *df* = 122) revealed that intentionality comprehension of intentional pain situations (*p* < 0.01) and neutral situations (*p* < 0.01) was higher than the comprehension of accidental pain situations. A significant interaction between group and condition [*F*_(2, 122)_ = 3.15, *p* < 0.05, η^2^ = 0.06] were also observed in intentionality judgments. *Post-hoc* analysis (Tukey's HSD, MS = 464.81, *df* = 179.92) revealed that bvFTD patients (hereafter referred to as patients) had significantly lower comprehension of the intentionality of accidental (*p* < 0.01) situations compared to controls. Intra-group comparisons using repeated-measures ANOVA showed significant differences in the intentionality comprehension among the 3 conditions in patients [*F*_(2, 64)_ = 4.75, *p* < 0.05, η^2^ = 0.14]. A *post-hoc* comparison (Tukey HSD, MS = 750.19, *df* = 64) revealed that intentionality comprehension of intentional (*p* < 0.05) and neutral situations (*p* < 0.05) was higher than the comprehension of accidental pain situations. In controls, there was a trend toward a better comprehension of intentional pain situations compared to the accidental ones [*F*_(2, 58)_ = 2.88, *p* < 0.06, η^2^ = 0.09].

Furthermore, a significant interaction between group and condition was observed in ratings of intention to hurt [*F*_(2, 122)_ = 16.44, *p* < 0.01, η^2^ = 0.21]. *Post-hoc* analysis (Tukey HSD, MS = 10.29, *df* = 23.72) showed that patients had higher ratings than controls for neutral (*p* < 0.05) and accidental (*p* < 0.01) situations. Intra-group comparisons using repeated-measures ANOVA showed significant differences in the intention to hurt ratings among the 3 conditions in both patients [*F*_(2, 64)_ = 94.45, *p* < 0.01, η^2^ = 0.77] and controls [*F*_(2, 58)_ = 316.54, *p* < 0.01, η^2^ = 0.91]. *Post-hoc* comparisons [patients:(Tukey HSD, MS = 6.19, *df* = 64), controls: (Tukey HSD, MS = 3.58, *df* = 58)] revealed that in both groups intention to hurt ratings for intentional pain situations were higher than for neutral (*p* < 0.01) and accidental (*p* < 0.01) situations. Furthermore, in both groups intention to hurt ratings for accidental pain situations were higher than for neutral situations (*p* < 0.01).

#### Affective components

A significant interaction between group an condition was observed in the empathic concern ratings [*F*_(2, 122)_ = 10.02, *p* < 0.01, η^2^ = 0.14]. *Post-hoc* analysis (Tukey HSD, MS = 10.69, *df* = 155.04) revealed that patients rated intentional pain situations lower (*p* < 0.05) than controls. Furthermore, controls showed higher empathic concern for intentional than accidental pain situations (*p* < 0.01). This difference was not observed in patients (*p* = 0.78).

#### Moral aspects

There was a significant interaction between group and condition in correctness ratings [*F*_(2, 122)_ = 513, *p* < 0.01, η^2^ = 0.07]. *Post-hoc* analysis (Tukey HSD, MS = 9.62, *df* = 170.63) showed that patients rated neutral situations as more incorrect than controls (*p* < 0.01). A significant interaction between group and condition [*F*_(2, 122)_ = 6.24, *p* < 0.01, η^2^ = 0.09] were also found in punishment ratings. *Post-hoc* analysis (Tukey's HSD, MS = 11.21, *df* = 127.24) showed that patients rated neutral (*p* < 0.01) situations higher than controls.

No RTs differences were observed between groups.

### Social cognition and EF

The OSCD and EF results are shown in Table 1 (see details in Supplementary Data). Regarding social cognition, patients showed lower performance on TASIT (as well as scores of sadness, fear and disgust recognition) and RMET scores than controls. No group differences were observed in SNQ scores. Regarding EF, patients showed a lower performance than controls on the IFS total score, cognitive flexibility, the Hayling test and the verbal phonological fluency task.

### Re-analysis of empathy data with social cognition measures as covariates

Group differences in the intentionality comprehension (accidental pain situations) remained significant after adjusting for OSCD [*F*_(1, 59)_ = 5.72, *p* < 0.05, η^2^ = 0.09]. Similarly, group differences in empathic concern for intentional pain situation were preserved [*F*_(1, 59)_ = 6.98, *p* < 0.05, η^2^ = 0.1]. Nonetheless, differences in intention to hurt ratings for neutral [*F*_(1, 59)_ = 0.95, *p* = 0.33, η^2^ = 0.01] and accidental situations [*F*_(1, 59)_ = 4.00, *p* = 0.06. η^2^ = 0.06] were not preserved after co-varying. Differences between groups in correctness [*F*_(1, 59)_ = 2.03, *p* = 0.15, η^2^ = 0.03] and punishment [*F*_(1, 59)_ = 2.63, *p* = 0.11, η^2^ = 0.04] ratings for neutral situations also disappeared (Figure 2).

### Re-analysis of empathy data with EF as covariates

Group differences in the intentionality comprehension (accidental pain situations) did not remain significant after adjusting for EF [*F*_(1, 53)_ = 2.24, *p* = 0.14, η^2^ = 0.03]. A significant effect of the Hayling test performance on accidental situations comprehension was observed [*F*_(1, 53)_ = 6.47, *p* < 0.05, η^2^ = 0.1].

Significant group differences in empathic concern ratings for intentional pain situations were preserved [*F*_(1, 53)_ = 16.53, *p* < 0.01, η^2^ = 0.24] after covariate analysis. However, differences in intention to hurt ratings for neutral [*F*_(1, 53)_ = 0.05, *p* = 0.81, η^2^ = 0.001] and accidental situations [*F*_(1, 53)_ = 1.07, *p* = 0.30, η^2^ = 0.01] were not preserved after co-varying, as well as correctness [*F*_(1, 53)_ = 0.12, *p* = 0.72, η^2^ = 0.001] and punishment [*F*_(1, 53)_ = 0.047, *p* = 0.82, η^2^ = 0.05] ratings for neutral situations (Figure 2).

### Is the empathy performance partially explained by EF, OSCD or general cognitive state?

**Figure 3:**
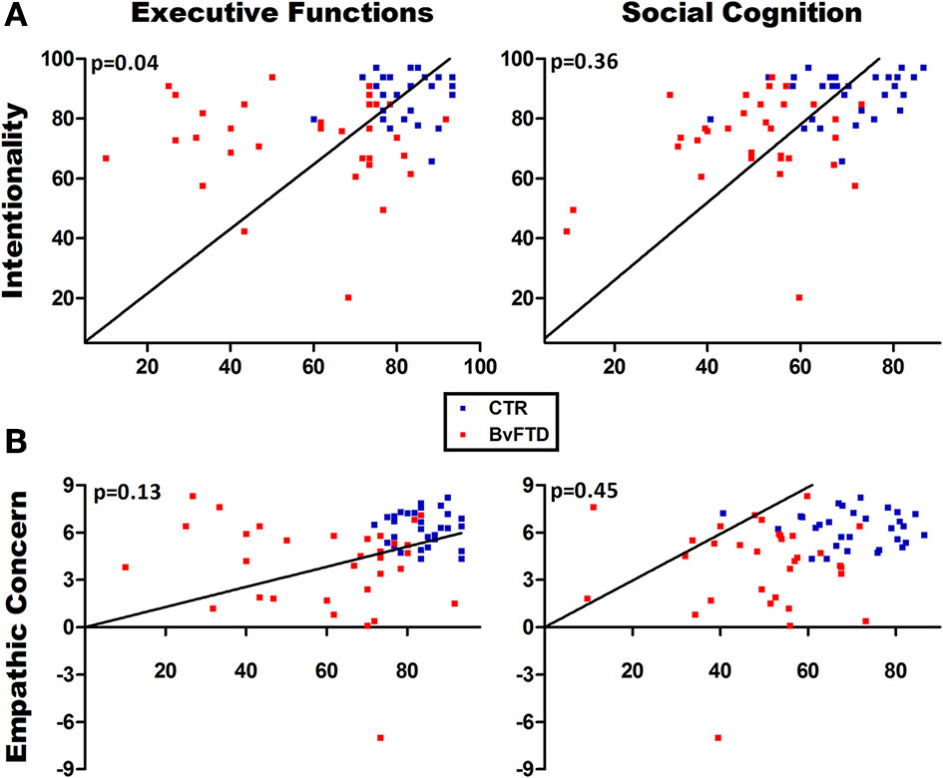
**Multiple regression analyses. (A)** Regression analysis using intentionality comprehension as the dependent variable. Executive functions significantly predicted the intentionality comprehension. **(B)** Regression analysis using empathic concern as the dependent variable. No significant associations were observed between empathic concern and social cognition or executive functions.

Figure 3 shows associations in multiple regression analyses indexing the role of EF and OSCD. A first model including the intentionality score as dependent variable [*F*_(4, 58)_ = 8.59, *p* < 0.01, R2 = 0.38] showed that EF (beta = 0.28, η^2^ = 0.06) and group (beta = −0.29, η^2^ = 0.06) predicted the intentionality comprehension, explaining 38% of the variance. We carried out a second model with empathic concern for intentional pain situations as dependent variable. This model [*F*_(4, 58)_ = 5.16, *p* < 0.01, *R*2 = 0.26] evidenced that group (but not EF or OSCD) was the only predictor (beta = 0.55, η^2^ = 0.16) associated with empathic concern ratings, explaining 26% of the variance.

To confirm this last result, we estimated three different multiple regression models considering the score of empathic concern for intentional pain situations as dependent variable and including independently group and all the EF and OSCD measures as predictors. The first model including all EF variables [*F*_(6, 56)_ = 3.60, *p* < 0.01, *R*2 = 0.22] showed that group (beta = 0.66, η^2^ = 0.23) was the only predictor associated to empathic concern. Similarly, the second model including all OSCD variables [*F*_(4, 58)_ = 3.90, *p* < 0.05, *R*2 = 0.30] evidenced that group (beta = 0.45, η^2^ = 0.11) was the only significant predictor. The third model including all EF and OSCD variables confirmed that group (beta = 0.54, η^2^ = 0.14) was the only predictor significantly associated with empathic concern [*F*_(9, 53)_ = 2.13, *p* < 0.05, *R*2 = 0.30] (see Table 2).

**Table 2:** **Coefficients of the multiple regression models of empathic concern**.

**Variables**	**Model I**		**Model II**		**Model III**	
	**β**	***p***	**β**	***p***	**β**	***p***
Group	0.66	0.0002	0.45	0.01	0.54	0.01
IFS total score	0.23	0.34			0.13	0.61
Phonological fluency	−0.18	0.29			−0.19	0.29
Design fluency	0.08	0.68			0.09	0.68
Cognitive flexibility (TMT-B)	0.28	0.11			0.34	0.16
Inhibitory control (Hayling test)	0.28	0.21			0.20	0.30
Emotion recognition (TASIT)			0.09	0.42	0.17	0.35
Theory of mind (RMET)			0.10	0.51	0.05	0.71
Social norms knowledge (SNQ)			−0.06	0.66	0.01	0.89

*IFS, INECO frontal screening; TMT, Trail Making Test; TASIT, The awareness of social inference test; RMET, Reading the mind in the eyes; SNQ, Social norms questionnaire*.

In brief, EF predicted the intentionality comprehension but not the empathic concern. Social cognition was not associated with any of the dependent variables. Empathic concern was not explained by any predictor.

To explore whether empathic concern depends on the general cognitive state or disease severity, we also compared the performance of patients with high (54%) and low (46%) MMSE scores (cut-off = 27). No group differences in empathic concern [*t*_(31)_ = 0.80, *p* = 0.42] were found, suggesting an early and primary involvement.

## Discussion

Although empathy deficits are considered a central feature and diagnostic criterion of bvFTD, no studies had directly explored the contribution of different empathy aspects and whether and how relevant factors such as EF and social cognition affect the empathic abilities of these patients. Our results replicate previous findings of EF (Torralva et al., 2009a,b) and social cognition (Gleichgerrcht et al., 2010; Possin et al., 2013) impairments in bvFTD [except for the lack of differences in SNQ scores (Possin et al., 2013)], that would be explained by population's cultural differences. Moreover, we provide evidence of a primary deficit in empathic concern that is not related to deficits in EF or OSCD. The identification and further assessment of the primary empathy deficits of bvFTD patients may be useful in the establishment of behavioral patterns and potentially in predicting the disease progression based on empathic concern levels.

### Differential impairments of empathy components

Impaired cognitive components (distinguishing accidental from neutral and intentional situations) were observed in patients. This is expected because empathy is a contextual phenomenon affected by stimulus ambiguity (Melloni et al., 2013). Contextual cues help to bias the intrinsic meaning of ambiguous targets (Bar, 2004; Amoruso et al., 2014), particularly regarding others in pain (Melloni et al., 2013) and social cognition (Ibanez et al., 2014a). According with a previous study in healthy subjects (Decety et al., 2012), our results show that intentionality comprehension of intentional pain situations was higher than the comprehension of accidental pain situations. This suggests that accidental pain situations are less clear and explicit, increasing the level of ambiguity and the demands in the attribution of the action's intentionality.

Moreover, patients with bvFTD have deficits in inferring the intentionality of others' actions (Gregory et al., 2002; Torralva et al., 2009a; Poletti et al., 2012), and in understanding ambiguous emotional scenes (Fernandez-Duque et al., 2010). Thus, our results seem to be consistent with the current view suggesting that these patients have deficits in processing contextual social cues (Neary et al., 1998; Ibanez and Manes, 2012). However, as the EPT employed here does not permit to disentangle whether patients have deficits in analyzing contextual social cues, further studies should strictly control for the context dependency levels of empathy tasks, including control conditions or experimental manipulation of contextual cues. In addition, it is worth to mention that the cognitive components of empathy assessed in this study have been associated to mentalizing (Zaki and Ochsner, 2012), a fundamental ability to empathize with others by considering their mental states. Impairments in this ability have also been reported in bvFTD patients (Downey et al., 2013; Cerami et al., 2014). Future studies using experimental paradigms for assessing empathy should include other cognitive aspects such as perspective taking.

Patients rated the intention to hurt for neutral/accidental situations higher than controls. In contrast, as reported by previous studies (Akitsuki and Decety, 2009; Decety et al., 2012), we found that intention to hurt ratings of healthy participants are greater for intentional than for accidental pain situations. Intentionality detection is a decisive step in determining whether an action was malicious (Decety et al., 2012). The inability to infer the intentions of others' actions may affect the intention to hurt ratings. Patients with bvFTD tend to overattribute bad intentions to the agent (Gregory et al., 2002; Kipps and Hodges, 2006), even if the action was unintentional.

Regarding affective components, bvFTD patients showed lower empathic concern ratings for intentional pain situations. Previous bvFTD studies (Lough et al., 2006; Rankin et al., 2006; Eslinger et al., 2011) have reported diminished levels of empathic concern as rated by relatives or caregivers. Thus, this characteristic appears to be a core component of bvFTD empathy impairments (see below).

On aspects related to moral evaluation, patients rated neutral situations more morally wrong than controls. However, neutral situations did not represent a wrong action. Again, these findings suggest deficits in inferring the intentionality of the action and in attributing bad intention, even when this was not the purpose. Moral reasoning relies on both affective and cognitive processes to integrate intentions and action consequences (Decety et al., 2012). In agreement with previous reports (Mendez et al., 2005; Lough et al., 2006; Mendez, 2006; Baez et al., 2014), our results suggest that moral reasoning is impaired in bvFTD.

Overall, the empathy profile of patients was characterized by impairments in cognitive, affective and moral aspects. Taking into account that intentionality detection is a decisive step in determining whether an action was malicious and ratings of intention to hurt are associated with ratings of punishment (Decety et al., 2012), our results suggest that deficits in the ability to infer the intentionality of another's actions seem to affect cognitive components and moral aspects. Conversely, empathic concern seems to be the only component primarily affected in bvFTD.

### Are empathy deficits explained by EF or OSCD?

Impairments in the cognitive components of bvFTD patients remained significant after adjusting for social cognition but disappeared after co-varying for EF. In line with this finding, previous studies (Rankin et al., 2005; Hansen, 2011; Ze et al., 2014) have been suggested a link between EF and empathy. Specifically, working memory (Ze et al., 2014), inhibitory control (Hansen, 2011; Ze et al., 2014), abstract reasoning and phonological fluency (Rankin et al., 2005) are particularly associated with self-report measures of cognitive empathy. Thus, inferring the others' intentions requires the inhibition of one's own perspective (Ruby and Decety, 2003; Samson et al., 2005). Furthermore, working memory is required to hold and manipulate cues from multiple sources of input, particularly in more complex social situations (Rankin et al., 2006; Meyer et al., 2012). During the EPT, accidental pain situations are less clear and explicit. Therefore, it is possible that the accurate recognition of these situations requires a higher EF demand.

Similarly, the significance of intention to hurt, correctness and punishment ratings also disappeared after co-varying for EF. These three empathy aspects are strongly dependent on the observer's interpretation of intention, and the EF profile seems to explain these deficits. In bvFTD (Lough et al., 2006; Eslinger et al., 2011), a relationship between cognitive components (rated by caregivers) and EF has been evidenced. The same group differences also disappeared after co-varying for social cognition, consistent with the fact that some aspects of empathy are related to emotion recognition (Martin et al., 1996; Besel and Yuille, 2010) and ToM (Shamay-Tsoory et al., 2007; Ibanez et al., 2013) abilities. Moreover, the deficits in moral aspects in bvFTD patients may be partially explained by an empathic loss in emotionally identifying with others (Mendez et al., 2005). Thus, emotion recognition and ToM deficits account for the abnormalities in cognitive and moral aspects of empathy observed in patients with bvFTD.

Differences in empathic concern for intentional situations remained significant after co-varying for both EF and OSCD. These results suggest that bvFTD patients have a core deficit in other-oriented emotional reactions to the misfortune of others. We performed multiple regressions to further explore which empathy aspects were primary affected. We choose a global score of the OSCD from TASIT and RMET because in this and previous studies (Kipps et al., 2009a; Torralva et al., 2009a) detected bvFTD impairments. The IFS was also selected as a predictor because this tool includes several EF subtests and detects bvFTD executive dysfunction (Torralva et al., 2009b). Multiple regression results showed that empathic concern was not predicted by EF or OSCD.

### Emphatic concern as the primary affectation of bvFTD

Taken together, our results suggest that empathic concern is the only component primarily affected in bvFTD that is neither related nor explained by EF/OSCD deficits or the general cognitive status. In contrast, deficits in cognitive and moral aspects of empathy seem to depend on other processes such as EF, emotion recognition or ToM.

The degree of discomfort (an affective component) was preserved in patients. Unlike empathic concern, the discomfort degree involves self-oriented feelings of personal unease when exposed to the suffering of others (Davis et al., 1994). Moreover, discomfort may produce an egoistic motivation to reduce one's own personal distress, whereas empathic concern may instigate an altruistic motivation to help the other. Thus, the other-oriented emotional response that produces a motivational state to increase the other's welfare was intrinsically affected in bvFTD, constituting the core of empathy impairments observed in these patients.

Theoretical approaches (Decety and Jackson, 2004) and empirical evidence (Rankin et al., 2006) agree that empathy relies on dissociable affective and cognitive components. Emotional components of empathy are foundational, while cognitive components are more complex and may depend upon other abilities (Rankin et al., 2006). Thus, diminished other-oriented emotional responses may be sufficient to produce the daily empathy impairments observed in bvFTD patients.

Neuroimaging studies of empathy (Carr et al., 2003; Rankin et al., 2006) highlight a network that includes the inferior and medial frontal cortex, amygdala, right somatosensory cortex, right temporal pole and insula; all brain areas usually affected in bvFTD (Rosen et al., 2002; Seeley et al., 2008; Couto et al., 2013). Moreover the subgenual cortex, as an ACC adjacent area may represent a point of interest for futures studies about empathy and its neurobiological bases in neurodegenerative diseases (Zahn et al., 2009). Overall, the brain atrophy pattern previously reported in bvFTD is consistent with the primary deficit in empathic concern observed in this study. Our findings open new pathways to investigate whether impairments in empathic concern could predict the atrophy pattern, behavioral changes, and the clinical profile of bvFTD. Although this is the first study in evidencing the empathic concern deficits usually reported by bvFTD relatives by means of an experimental method, our patients were assessed only with routine MRI recordings. Further volumetric and fMRI studies may provide additional insights about the relationship among the location of atrophy and the associated pattern of empathy impairments. An inter-level social neuroscience approach (Ibanez et al., 2014a) combining the study of social behavior, neural networks, and the interactions between social behaviors and social cognition would help to provide a better understanding of bvFTD (Ibanez et al., 2014b). This novel approach would allow psychiatrists and neurologists to contribute a powerful multidisciplinary and transdisciplinary approach (Maj, 2012), that would be both clinically and theoretically relevant to major advances in contemporary neuropsychiatry.

From a clinical perspective, given that adequate empathic functioning is an important element of higher social functioning (Rankin et al., 2005), such an impairment should be considered in the assessment and treatment of bvFTD, as well as during cognitive-affective interventions (Ibanez et al., 2014c). Furthermore, one of the strengths of the current study is its reliance on an ecological design that is more appropriate than self-report questionnaires. However, future studies should assess whether empathy aspects evaluated by experimental tasks are related to the components measured by classical self-report questionnaires such as the Index of Interpersonal Reactivity.

It is well-known that even frontal patients are impaired in their everyday lives. It is difficult to detect impairments with traditional tests because standard and decontextualized neuropsychological assessments introduce sufficient external structure to suppress some behavioral tendencies (Mesulam, 1986). Besides traditional methods for assessing cognitive deficits following frontal lobe damage typically does not measure the full range of deficits that can occur. In particular, rostral prefrontal cortex supports functions which are not routinely assessed yet are crucial to competent everyday life performance (Burgess et al., 2009). Remarkably, the task employed here detected experimentally (Lough et al., 2006; Eslinger et al., 2011) early empathy deficits in bvFTD patients. The convergence between observations in experimental, clinical and everyday life settings highlights the importance of considering empathic concern impairments as a core symptom of bvFTD. These results emphasize the value of using tasks involving real-life social scenarios (Torralva et al., 2009a; Ibanez and Manes, 2012) as evidenced by their greater sensitivity in the clinical assessment of neuropsychiatric populations. Moreover the current findings suggest that social cognition assessment, particularly the evaluation of empathy, should be part of the clinical screening for dementia. Future studies should explore differences between bvFTD and other forms of dementia and test whether empathy could predict the likelihood of bvFTD. A more subtle understanding of these complex cognitive deficits in bvFTD will improve assessment in the clinical setting and may allow for the development of rational cognitive stimulation strategies.

### Conflict of interest statement

This study was partially supported by Consejo Nacional de Investigaciones Científicas y Técnicas (CONICET) and Fundación Instituto de Neurología Cognitiva (INECO) Foundation. Dr. Ibanez reports having received research funding from CONICYT/FONDECYT Regular (1130920 and 1140114), PICT 2012-0412, and PICT 2012-1309. Dr. Diana Matallana reports having received research funding from COLCIENCIAS (371-2011). The other authors report no disclosures relevant to this manuscript. The authors declare no competing financial interests.
