# Supplementary material for: Primary empathy deficits in frontotemporal dementia
Source: Front Aging Neurosci. 2014 Oct 10;6:262. doi: 10.3389/fnagi.2014.00262 (PMC4193328; doi:10.3389/fnagi.2014.00262)
Supplement: Supplementary file 2 [file DataSheet1.DOC]

***Supplementary Material***

**Primary empathy deficits in frontotemporal dementia**

Sandra Baez^1,2,3,4 Ω^, Facundo Manes^1,2,3,10^,David Huepe^2,8^, Teresa Torralva^1,2^, Natalia Fiorentino^1,2^, Fabian Richter^5^, Daniela Huepe^2,8^ ,Jesica Ferrari^1^, Patricia Montañes^6^, Pablo Reyes^6^, Diana Matallana^6^, Nora Silvana Vigliecca^3,7^ Jean Decety^9^, and Agustin Ibanez^1,2,3, 10 *^

1 Institute of Cognitive Neurology (INECO) & Institute of Neuroscience, Favaloro University, Buenos Aires, Argentina.

2 UDP-INECO Foundation Core on Neuroscience (UIFCoN), Diego Portales University, Santiago, Chile.

3 National Scientific and Technical Research Council (CONICET), Argentina.

4 Pontifical Catholic University of Argentina, Buenos Aires, Argentina.

5 Department of Psychology, University of Cologne, Cologne, Germany.

6 Universidad Javeriana, Facultad de Medicina. Instituto de Envejecimiento, departamento de Psiquiatría y Salud Mental. Centro de memoria y Cognición Intellectus, Hospital San Ignacio.

7 Instituto de Humanidades (IDH) de la Facultad de Filosofía y Humanidades, Universidad Nacional de Córdoba.

8 Laboratory of Cognitive and Social Neuroscience, Universidad Diego Portales, Santiago, Chile.

9 Department of Psychology and Department of Psychiatry and Behavioral Neuroscience, University of Chicago, Chicago, IL, USA.

10 Australian Research Council (ACR) Centre of Excellence in Cognition and its Disorders, Sidney, Australia.

*** Corresponding author:**

Agustin Ibanez (aibanez@ineco.org.ar) Laboratory of Experimental Psychology & Neuroscience (LPEN), Institute of Cognitive Neurology (INECO) & CONICET. Pacheco de Melo 1860, Buenos Aires, Argentina, Postal code 1126. Phone/Fax: +54 (11) 4807-4748.

Ω This work is part of the PhD dissertation [Sandra Baez] ongoing by the author at Pontifical Catholic University of Argentina

**Supplementary Data**

**Patients with possible differential diagnosis**

We verified individually that all patients included in this study met the revised criteria for probable bvFTD . Although all of them met these criteria, two patients had a questionable diagnosis. One of the patients showed bilateral anterior temporal atrophy in MRI and evident progression of behavioral symptoms. However, the cognitive profile has remained stable over a period of three years. Furthermore, this patient exhibited empathic concern ratings over the mean level of bvFTD group. Taking into account these features, there is a chance that this patient may qualify for a diagnosis of the phenocopy syndrome .

Regarding the other patient, the late-onset of the typical bvDFT behavioral symptoms and the presence of depressive symptoms, hyperthymic temperament and episodic affective symptoms many years before the onset of the disease, make us contemplate the differential diagnosis of late-onset bipolar disorder . The mentioned symptoms do not meet strict criteria for depressive or manic episodes and we did not have the possibility to do a long-term follow up of the patient to exclude bipolar disorder diagnosis.

In spite of these possible differential diagnoses, we did not exclude these two patients of our sample because this did not modify the results of the statistical analyses.

**Results**

**Social cognition and EF results**

Regarding emotion recognition, TASIT total scores revealed a lower performance in bvFDT patients (*F*(1,64) = 42.44, *p <* 0.01). The per category analysis showed significant differences between groups (*F*(4, 248) = 2.73, *p <* 0.05). A post-hoc analysis (Tukey HSD, *MS* = 0.62, *df* =280.15) revealed that bvFTD patients had difficulty with sadness (*p <* 0.01), fear (*p <* 0.01) and disgust (*p <* 0.01) categorization. No significant differences were observed for anger (p = 0.20) or surprise (p = 0.80) categorization.

In ToM assessment BvFTD patients showed lower scores than controls on the RMET (*F*(1, 65) = 33.26, *p <* 0.01). No significant differences between groups were observed in the break (*F*(1, 65) = 0.40, *p =* 0.52) and over-adhere (*F*(1, 65) = 1.23, *p =* 0.27) scores of the SNQ.

With respect to EF, bvFTD patients showed a lower performance than controls on the IFS total score (*F*(1,65) = 38.35, *p <* 0.01). BvFTD group also had lower scores on the cognitive flexibility measures, including the TMT-B (*F*(1,65) = 18.18, *p <* 0.01) and the switching design fluency task (*F*(1,65) = 50.07, *p <* 0.01). Furthermore, bvFTD patients exhibited a lower performance than controls on the Hayling test (*F*(1,65) = 23.88, *p <* 0.01) and the verbal phonological fluency task (*F*(1,65) = 42.93, *p <* 0.01).

1. **References**

Azorin, J.M., Kaladjian, A., Adida, M., and Fakra, E. (2012). Late-onset bipolar illness: the geriatric bipolar type VI. *CNS Neurosci Ther*. **18**:3. doi: 10.1111/j.1755-5949.2011.00255.x.

Kipps, C.M., Hodges, J.R., and Hornberger, M. (2010). Nonprogressive behavioural frontotemporal dementia: recent developments and clinical implications of the 'bvFTD phenocopy syndrome'. *Curr Opin Neurol*. **23**:6. doi: 10.1097/WCO.0b013e3283404309.

Rascovsky, K., Hodges, J.R., Knopman, D., Mendez, M.F., Kramer, J.H., Neuhaus, J., Van Swieten, J.C., Seelaar, H., Dopper, E.G., Onyike, C.U., Hillis, A.E., Josephs, K.A., Boeve, B.F., Kertesz, A., Seeley, W.W., Rankin, K.P., Johnson, J.K., Gorno-Tempini, M.L., Rosen, H., Prioleau-Latham, C.E., Lee, A., Kipps, C.M., Lillo, P., Piguet, O., Rohrer, J.D., Rossor, M.N., Warren, J.D., Fox, N.C., Galasko, D., Salmon, D.P., Black, S.E., Mesulam, M., Weintraub, S., Dickerson, B.C., Diehl-Schmid, J., Pasquier, F., Deramecourt, V., Lebert, F., Pijnenburg, Y., Chow, T.W., Manes, F., Grafman, J., Cappa, S.F., Freedman, M., Grossman, M., and Miller, B.L. (2011). Sensitivity of

revised diagnostic criteria for the behavioural variant of frontotemporal dementia. *Brain*. **134**:Pt 9. doi: 10.1093/brain/awr179.
